# Flow Cytometry Combined With Single Cell Sorting to Study Heterogeneous Germination of *Bacillus* Spores Under High Pressure

**DOI:** 10.3389/fmicb.2019.03118

**Published:** 2020-01-21

**Authors:** Yifan Zhang, Alessia I. Delbrück, Cosima L. Off, Stephan Benke, Alexander Mathys

**Affiliations:** ^1^Sustainable Food Processing Laboratory, Institute of Food, Nutrition and Health, Department of Health Science and Technology, ETH Zürich, Zurich, Switzerland; ^2^Cytometry Facility, University of Zurich, Zurich, Switzerland

**Keywords:** bacterial spore, high pressure, flow cytometry, fluorescence-activated cell sorting, germination, heterogeneous, inactivation, *Bacillus*

## Abstract

Isostatic high pressure (HP) of 150 MPa can trigger the germination of bacterial spores, making them lose their extreme resistance to stress factors, and increasing their susceptibility to milder inactivation strategies. However, germination response of spores within a population is very heterogeneous, and tools are needed to study this heterogeneity. Here, classical methods were combined with more recent and powerful techniques such as flow cytometry (FCM) and fluorescence activated cell sorting (FACS) to investigate spore germination behavior under HP. *Bacillus subtilis* spores were treated with HP at 150 MPa and 37°C, stained with SYTO16 and PI, and analyzed via FCM. Four sub-populations were detected. These sub-populations were for the first time isolated on single cell level using FACS and characterized in terms of their heat resistance (80°C, 10 min) and cultivability in a nutrient-rich environment. The four isolated sub-populations were found to include (1) heat-resistant and mostly cultivable superdormant spores, i.e., spores that remained dormant after this specific HP treatment, (2) heat-sensitive and cultivable germinated spores, (3) heat-sensitive and partially-cultivable germinated spores, and (4) membrane-compromised cells with barely detectable cultivability. Of particular interest was the physiological state of the third sub-population, which was previously referred to as “unknown”. Moreover, the kinetic transitions between different physiological states were characterized. After less than 10 min of HP treatment, the majority of spores germinated and ended up in a sublethally damaged stage. HP treatment at 150 MPa and 37°C did not cause inactivation of all geminated spores, suggesting that subsequent inactivation strategies such as mild heat inactivation or other inactivation techniques are necessary to control spores in food. This study validated FCM as a powerful technique to investigate the heterogeneous behavior of spores under HP, and provided a pipeline using FACS for isolation of different sub-populations and subsequent characterization to understand their physiological states.

## Introduction

### Isostatic High Pressure Processing as a Basis for Mild Spore Control Strategies

Spore-forming bacteria, mainly represented by the genera *Bacillus* and *Clostridium*, are ubiquitous in nature. As a consequence, they inevitably enter the food chain, and potentially cause food spoilage and food-borne illnesses, leading to economic losses and increased public health risks (Setlow and Johnson, 2007; Reineke et al., 2013b; Wells-Bennik et al., 2016). These bacteria can form spores when the environmental conditions become unfavorable for their survival. These spores are extremely resistant to heat, dehydration, and chemical or physical stresses, and thus a major challenge in food decontamination processes (Setlow, 2006, 2007; Setlow and Johnson, 2007; Patrignani and Lanciotti, 2016; Zhang et al., 2018). Depending on food composition and storage conditions, surviving spores can eventually germinate and grow out, and cause food quality and/or safety problems. For food products where absence of spores is essential, intensive wet heat treatment is usually applied to directly inactivate spores (Storz and Hengge, 2010; Georget et al., 2013). However, such procedures often cause unwanted losses in food quality. Therefore, effective and gentle alternative non-thermal spore control strategies are of high interest (Storz and Hengge, 2010; Sevenich and Mathys, 2018; Zhang et al., 2018). The reader is referred to Reineke and Mathys (2020) for an extensive review of different spore inactivation pathways by emerging technologies.

A more gentle approach to control spores aims on not directly inactivating the dormant spores but rather using a so called germination-inactivation strategy (Collado et al., 2004; Abee et al., 2011; Lovdal et al., 2011; Zhang and Mathys, 2019). This strategy is based on the well-studied phenomenon that dormant spores lose their resistance after germination, and thus become sensitive toward additional mild decontamination procedures (Collado et al., 2004; Setlow, 2006; Abee et al., 2011; Lovdal et al., 2011). Therefore, spore germination was studied extensively in the last decades. Spore germination can be triggered by different stimuli, including nutrients, Ca^2+^-dipicolinic acid (Ca^2+^-DPA), and isostatic high pressure (HP) (Gould, 1970; Setlow, 2003; Gould, 2006; Baier et al., 2011; Reineke et al., 2013b; Sevenich and Mathys, 2018). From a practical perspective, HP offers clear advantages over other germination-triggering strategies: food products can be treated more homogenously, addition of chemicals like Ca^2+^-DPA are not necessary, and germinated spores and other vegetative cells can be inactivated simultaneously (Gould and Sale, 1970; Knorr et al., 1998; Georget et al., 2014; Doona et al., 2016; Sevenich and Mathys, 2018; Zhang and Mathys, 2019). As a promising non-thermal spore-controlling method, HP also retains food quality better than the state-of-the-art heat sterilization in terms of nutritional value, color and other sensorial attributes (Martinez-Monteagudo et al., 2014; Sevenich and Mathys, 2018).

Considering the high potential of HP technology for spore control, researchers have investigated the mechanisms involved in HP-induced germination in the past decades (Doona et al., 2014; Georget et al., 2014a; Luu et al., 2015). High pressure can be applied in continuous dynamic (Georget et al., 2014b; Dong et al., 2015) and discontinuous isostatic modes (Knorr et al., 2010), where relevant HP induced germination mechanisms were mainly observed for discontinuous isostatic treatment (Sevenich and Mathys, 2018). Different germination mechanisms were found to dominate within different pressure, temperature and time ranges (Wuytack et al., 1998; Paidhungat et al., 2002; Black et al., 2005, 2007). At lower pressures between 100 and 200 MPa at around 30–50°C, nutrient germinant receptors are activated, and the germination process is similar to nutrient-triggered germination (Wuytack et al., 1998; Paidhungat et al., 2002; Black et al., 2005). On the other hand, at higher pressures of between 500 and 600 MPa at < 60°C, Ca^2+^-DPA channels are directly opened (Paidhungat et al., 2002; Black et al., 2007; Reineke et al., 2013b). At higher pressures (>600 MPa) and higher temperatures (>60°C) spores are inactivated without triggering physiological processes involved in spore germination (Margosch et al., 2006; Reineke et al., 2011). The reader is referred to Reineke et al. (2013b) for a comprehensive review of different *Bacillus* spore germination and inactivation pathways under HP. Despite the efforts to elucidate the mechanistic pathways and potential applications of HP for spore control in food, successful application of HP-triggered germination-inactivation strategies is still hindered by barriers.

The primary reason preventing success, is the inability of HP in germinating and/or inactivating all bacterial spores. Similar to other germination stimuli, HP cannot trigger germination of all spores in a bacterial population. Bacterial spores are notorious for their unpredictable germination responses, resulting in heterogeneous behavior within a population (Setlow, 2003; Luu et al., 2015; Wells-Bennik et al., 2016; Zhang and Mathys, 2019). A fraction of spores, termed superdormant (SD) spores, tend to stay dormant or germinate extremely slowly compared to the rest of the population upon facing germination stimuli (Setlow, 2003; Ghosh and Setlow, 2009; Wells-Bennik et al., 2016). This heterogeneous germination behavior is most likely a population survival strategy: the risk of complete eradication of the population upon rapid environmental changes is lowered by keeping some spores dormant (Setlow et al., 2017). This strategy is fascinating and evolutionarily smart, yet heterogeneity in germination still poses severe industrial challenges for spore elimination using germination-inactivation based strategies. Spores that remain dormant even after HP treatment, in this case termed high-pressure superdormant (HPSD) spores, maintain their resistance, and therefore survive the subsequent mild inactivation process. Understanding heterogeneous spore germination behavior under HP is there essential in order to overcome this challenge and further develop HP-based germination-inactivation strategies.

### Methods to Study Heterogeneous Spore Germination

Several methods were developed previously for studying spore germination based on physiological changes that occur during germination. For example, phase-contrast microscopic examination and optical density drop evaluation are based on refractivity change, plate count with and without heat treatment is based on heat resistance loss during germination, and different methods were developed to measure Ca^2+^-DPA release during germination (Zernike, 1955; Hashimoto et al., 1969; Wuytack et al., 1998; Reineke et al., 2011; Kong et al., 2014; Setlow, 2014). However, most of these classical methods have low throughput and the obtained information only reflects the average population under investigation without taking into account heterogeneity within the population. These methods are therefore not ideal for studying heterogeneous spore germination (Reineke et al., 2013b; Zhang and Mathys, 2019). Considering the loss of important information concerning germination heterogeneity in population-level studies, researchers have investigated other techniques to study germination on a single cell level (Margosch et al., 2004; Chen et al., 2006; Zhang et al., 2012; Pandey et al., 2013; van Melis et al., 2014; Trunet et al., 2017). Examples include dilution (Margosch et al., 2004), Raman spectroscopy with laser tweezers (Chen et al., 2006; Kong et al., 2011), and flow cytometry (FCM) (Mathys et al., 2007; Wells-Bennik et al., 2016).

Here, we focused on validation and further development of a FCM-based method to investigate spore germination heterogeneity under HP. FCM is a powerful technology that allows rapid determination of structural and physiological status of individual spores from a heterogeneous population. In FCM, spores with and without fluorescent markers are aligned in a fluid stream, and pass through the foci of multiple laser beams one by one. The scattered and fluorescent light resulting from each single cell are collected and analyzed to obtain information on, e.g., cell viability, membrane permeability or metabolic activity, depending on the fluorescent probes used. This information can be further used to differentiate cells based on their structural or physiological states. Beyond single cell analysis, FCM also offers further advantages over classical methods for studying spore germination. For example, FCM allows the detection of rare events with higher statistical significance and throughput (Ueckert et al., 1995; Mathys et al., 2007; Karava et al., 2019). Another significant advantage of FCM is the isolation of interesting targets on single cell level for further investigation when coupled with a cell sorter (Ambriz-Avina et al., 2014; Karava et al., 2019). FCM has thus been used increasingly for studying spore germination in general. However, only very few studies used FCM to study spore germination under HP (Black et al., 2005; Mathys et al., 2007; Baier et al., 2011; Reineke et al., 2013a; Borch-Pedersen et al., 2017).

### Current State and Challenges of Using Flow Cytometry to Study Spore Germination

Previous studies on HP-treated *Bacillus* spores reported the usage of nucleic acid binding stains including the membrane-permeable and -impermeable stains SYTO16 and propidium iodide (PI), respectively, to distinguish between dormant, germinated and potentially inactivated spores (Black et al., 2005, 2007; Mathys et al., 2007; Reineke et al., 2013a; Borch-Pedersen et al., 2017). The underlying assumption here is that dormant spores should not or only very poorly be stained by either SYTO16 or PI. This is due to low inner membrane permeability, the presence of the cortex, and the presence of DNA-binding proteins which block access of the stains to the DNA (Ragkousi et al., 2000; Black et al., 2005; Kong et al., 2010). However, following Ca^2+^DPA release, degradation of DNA binding proteins and the degradation of the spore cortex during germination, germinated spores can be stained by the membrane-permeable dye SYTO16 and emit green fluorescence (Black et al., 2005; Kong et al., 2010). The membrane-impermeable red fluorescent dye PI only stains spores with compromised cell membranes, and thus indicates inactivation (Stiefel et al., 2015). Therefore, staining with SYTO16 and PI theoretically allows researchers to distinguish between dormant spores (PI- and SYTO16-negative), germinated intact spores (SYTO16-positive, PI-negative) and spores with compromised membranes (PI-positive). In a previous study investigating HP-treated *Bacillus subtilis* and *Bacillus licheniformis* spores (150 MPa, 37°C), at least one additional sub-population with intermediate SYTO16-intensity was reported. This sub-population was found to be predominant after a 10–20 min HP-treatment, and referred to as “unknown”, since its physiological state was poorly understood (Mathys et al., 2007; Reineke et al., 2013a; Borch-Pedersen et al., 2017). FCM results from previous studies were mostly verified by correlating or qualitatively comparing findings with those obtained by using classical methods such as Ca^2+^-DPA release, phase-contrast microscopy or viable plate count (Black et al., 2005; Mathys et al., 2007; Baier et al., 2011; Borch-Pedersen et al., 2017). However, as mentioned above, population heterogeneity cannot be analyzed in detail via classical methods. Therefore, isolation of each sub-population is necessary in order to truly understand the differences between their physiological states. Moreover, previous FCM studies with HP mostly did not provide sufficient details on important parameters and necessary controls, which limited reproducibility. It is also important to report the applied gating scheme for an objective presentation of data (Alvarez et al., 2010), yet this aspect was mostly overlooked in previous FCM studies on spore germination.

Considering the reproducibility issues and open questions regarding the physiological state of certain sub-populations in FCM analyses, here we provide a detailed and comprehensive protocol for investigation of HP-treated spores via FCM, as well as a systematic validation of its biological interpretation. This is the first study reporting the application of fluorescence-activated cell sorting (FACS) for isolation of individual sub-populations after HP treatment and investigation of their physiological states including cultivability and heat resistance. Sorting was performed on sub-population as well as single cell levels in order to study heterogeneity between and within sub-populations. Overall, this research provides a better understanding of heterogeneous spore behavior under HP, and contributes to further investigations of HP germination mechanisms and development of mild HP-based spore germination-inactivation strategies.

## Materials and Methods

### *Bacillus* Strain and Spore Preparation

The strain *B. subtilis* PS533 used in this study is an isogenic derivate of strain PS832, a laboratory derivative of 168. *B. subtilis* PS533 carries the plasmid pUB110 encoding resistance to kanamycin (Setlow and Setlow, 1996). Overnight cultures of *B. subtilis* PS533 in tryptic soy broth (TSB) with 10 μg/ml kanamycin were streaked onto Difco Sporulation Medium (DSM, pH 7.6) (Nicholson and Setlow, 1990), and incubated for 4–5 days at 37°C. The sporulation progress was monitored daily using a phase-contrast microscope (DM6, Leica Microsystems, Wetzlar, Germany). Spores were harvested when the ratio of phase-bright population reached > 95% and washed at least four times at 6000 × *g* for 10 min with sterile MilliQ water the day of harvest. Spores were further washed daily in the morning and afternoon the first week after harvesting. As determined by phase contrast microscopic examination, spores used in this work were 98% phase-bright dormant spores free of germinated spores, unreleased spores or cell debris and agglomerates. Spores were stored in the dark at 4°C in sterile MilliQ water, and washed on a biweekly basis.

### Sample Preparation and High Pressure Treatment

Prior to HP treatment, spores in sterile MilliQ water were diluted in 0.1 μm filtered (Minisart^®^, Germany) *N*-(2-acetamido)-2-aminoethanesulfonic acid (ACES, ThermoFisher, Kandel, Germany) buffer solution at pH 7.0 to a final concentration of 50 mM ACES, and a final spore concentration of approximately 10^9^ spores/ml. Spores were transferred into cryotube vials (Nunc A/S, Roskilde, Denmark), and sealed with a sealing tube (Nunc CryoFlex Tubing, Nunc A/S, Roskilde, Denmark) to prevent leakage during HP treatment. Spores were treated at 150 MPa and 37°C using a dual vessel high-pressure unit (modified Model U111, Unipress, Warsaw, Poland) for 3, 10, 20, or 40 min. One vessel served as the sample treatment chamber, and the other one as control to monitor the temperature. The control vessel was equipped with a K-type thermocouple mounted at the geometrical center of the tube, which allowed monitoring of the temperature that is representative of that within the sample tube. The vessels were immersed into a water bath (Huber CC410, Offenburg, Germany), and *bis*(2-ethylhexyl)sebacate (Sigma-Aldrich, Steinheim, Germany) was used as pressure-transmitting medium. The pressure and temperature at the inner center of the tubes were continuously monitored to ensure isothermal and isobaric conditions are maintained during dwell time. Pressure build-up and decompression rates were approximately 7 and 12 MPa/s, respectively. A typical pressure/temperature time profile is given in the Supplementary Material. After HP treatment, samples were immediately removed from the vessel, and placed on ice. An untreated control was prepared for each experiment, and kept on ice until used in further analyses.

### Flow Cytometry Analysis

Prior to FCM analysis, aliquots of untreated as well as HP-treated spores were diluted to a final concentration of approximately 10^7^ spores/ml using 0.1 μm filtered MilliQ water. Different concentrations of SYTO16 and PI as well as staining times were investigated to optimize the separation of different sub-populations in the treated samples. For best separation, samples were stained with SYTO16 to a final concentration of 0.1 μM (Molecular Probes, Leiden, Netherlands, SYTO16 was dissolved in DMSO, Fisher Chemical, United Kingdom) for 12 min before adding PI to a final concentration of 1.5 μM (Molecular Probes, Leiden, Netherlands, PI was dissolved in 0.1 μm filtered MilliQ) and incubated for another 4 min. Samples were kept at approximately 23°C in the dark to prevent bleaching of the stain, and analyzed immediately after a total of 16 min staining time.

Flow cytometry analyses were performed using a BD LSRFortessa^TM^ cytometer (BD Biosciences, Franklin Lakes, NJ, United States). It is worth mentioning that a flow cytometer with high sensitivity is necessary to detect particles of small size such as spores. The overall sensitivity is dependent on several factors including laser power and the sensitivities of various detectors. The green fluorescence of SYTO16, indicative of spore cortex hydrolysis, was induced by a 488 nm continuous wave laser at a power of 55 mW, and collected through a 530/30 band-pass filter. The red fluorescence of PI, indicative of inner membrane damage, was induced by a 561 nm continuous wave laser at a power of 53 mW, and collected through a 610/20 band-pass filter. FacsFlow^TM^ (BD Biosciences, Franklin Lakes, NJ, United States) was used as sheath fluid. Sample acquisition was carried out at “low” sample flow rate (approximately 12 μl/min), yielding an event rate of approximately 8000 events/s. A total of 15000 events were acquired per sample. Data were acquired using BD FACSDiva software (BD Biosciences, Franklin Lakes, NJ, United States), and the height (H) and area (A) parameters were recorded for each channel.

Four controls were run for each sample: (1) 0.1 μm filtered MilliQ water to monitor the background signals, (2) untreated and unstained spores to control for sample homogeneity, (3) untreated and stained spores to exclude signals from the untreated sample itself in PI and SYTO16 channel, and (4) treated and unstained spores to exclude autofluorescence from the treated sample. Compensation was not necessary in our setting since no signal from PI was detected in the designated SYTO16 channel and vice versa.

**FIGURE 1:**
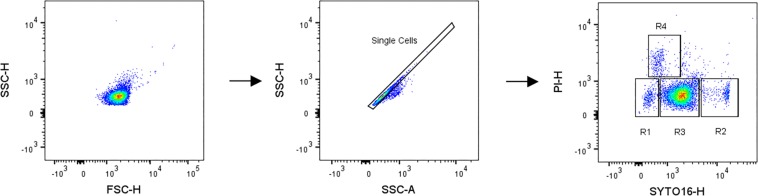
Gating scheme applied for sequential flow cytometry analysis of high pressure treated *Bacillus subtilis* spore suspensions on pseudocolor density plots on a biexponential scale. High pressure treated spore suspensions were stained with SYTO16 and PI, gated for single cells to exclude cell aggregates or multiple cells measured simultaneously (SSC-A, side scatter area vs. SSC-H, side scatter height) and finally gated for their fluorescent signals intensities (SYTO16-H vs. PI-H).

The data from the acquired FCS files were analyzed using FlowJo software (FlowJo LLC, Ashland, OR, United States). All samples were gated using the same gating tree and gate positions: (1) side scatter area (SSC-A) vs. side scatter height (SSC-H) to gate for single cells and exclude cell aggregates or multiple cells measured simultaneously, and (2) SYTO16 vs. PI to gate for their fluorescent signal intensities (Figure 1). Data are displayed as pseudocolor density plots on a biexponential scale (Parks et al., 2006). An untreated and stained control sample including dormant spores was used as reference in order to set the position of gate R1. The other gates were set based on histograms showing positions of optimal separation of two neighboring groups.

The technical variance of the developed staining protocol and FCM analysis was investigated with three independently-treated spore samples (*n* = 3) at 150 MPa, 37°C, 10 min and three technical replicates each. Furthermore, the influence of time between HP treatment and FCM analysis was investigated via three independent pressure treatments (*n* = 3). For this purpose, spores were HP-treated for 3 and 10 min, and FCM analysis was conducted at 25, 90, 150, and 180 min after decompression. Samples were stored at 4°C in between analyses.

### Comparison of Results From FCM, Plate Count and Phase-Contrast Microscopy on the Overall Population

Flow cytometry analysis results were quantitatively validated by using the viable plate count method by determining the percentage of HPSD and inactivated spores in the overall population, and qualitatively using phase-contrast microscopy.

For viable plate count, all HP-treated spores were plated on tryptic soy agar (TSA) with or without prior heat treatment at 80°C for 10 min, and incubated for 16 to 48 h at 37°C. Spores surviving heat treatment were defined as HPSD spores. The percentage of inactivated cells were determined by comparing the number of surviving cells before and after HP without heat treatment. Experiments were performed three times independently (*n* = 3). Results were expressed as mean value ± standard deviation.

HP-treated samples were further investigated qualitatively under a phase-contrast microscope (DM6, Leica Microsystems, Wetzlar, Germany) to validate FCM analysis results. Due to refractive index changes, germinated spores appear phase-dark and HPSD appear phase-bright (Hashimoto et al., 1969).

### FACS Sorting

A BD FACSAria^TM^ III (BD Biosciences, Franklin Lakes, NJ, United States) device was used to sort sub-populations after HP treatment. The optical set-up was the same as for the FCM analyzer. SYTO16 was excited by a 488 nm laser, and the emission signal was collected through a 530/30 filter. PI was excited by a 561 nm laser, and the emission signal was collected through a 610/20 filter. Gibco^TM^ PBS (1x, pH 7.4, Thermo Fisher Scientific, Waltham, MA, United States) was used as a sheath fluid. Spore samples were diluted to a final concentration of approximately 10^7^ spores/ml with 0.1 μm filtered 50 mM ACES at pH 7. Samples were acquired at the lowest flow rate (1), resulting in approximately 6000 event/s. Sorting was performed using a 70 micron nozzle at 87 kHz. The drop delay was set manually using BD FACS^TM^ Accudrop Beads (BD Biosciences, Franklin Lakes, NJ, United States) before the experiment. The sorting accuracy at the set drop delay was checked by sorting a target group of a stained sample into a FACS tube, and performing re-analysis with the sorted sample. Further experiments were only performed when the re-analysis showed that >95% sorted cells were from the target group. The same gating scheme as described in Section “Flow Cytometry Analysis” was applied for sorting. Preliminary work showed that the overall cultivability of HP-treated spores in ACES buffer decreased over time. Therefore, all sorting was performed immediately after decompression.

Treated spores were sorted into 96-well plates (SPL Life Sciences, Pocheon, South Korea) filled with TSA. The sorting precision was set to “single cell” (yield mask: 0; purity mask: 32; phase mask: 16) to maximize the purity and counting accuracy. After sorting, the 96-well agar plates were incubated at 37°C, and the colony formation in each well was checked after 24 and 48 h. The cultivability was calculated by comparing number of wells with a CFU with the total number of wells. The index sorting function of the sorter was also activated when sorting into 96-well plates. Using this function, information on parameters like forward scatter (FSC), side scatter (SSC), SYTO16 and PI signals were recorded for each event that was sorted into each specific well. This way, information can be traced back to specific wells of interest. Information on “cultivable” and “non-cultivable” sorted events and their respective SYTO16 and PI signals were then combined, and plotted to visualize the distribution of cultivability.

#### Influence of Staining on Cultivability of Sorted Cells

SYTO16- and PI-staining were used in this study to distinguish different sub-populations from each other after HP treatment. Both stains bind to the DNA, and thus may influence the cultivability of stained and sorted cells. Therefore, it is important to understand whether the staining influences the physiological states of the sorted cells. For this purpose, HP-treated (150 MPa, 37°C, 10 min) samples were single cell sorted into 96-well TSA plates with and without SYTO16- and PI-staining. For both stained and unstained conditions, three 96-well plates were sorted (*n* = 3), and experiments were repeated three times on different days independently (*n* = 3). Results were expressed as mean value ± standard deviation.

#### Physiological States of Spores After HP Treatment

In order to obtain an overview of the influence of the HP treatment on the physiological state of spores, the entire population from a HP-treated sample (150 MPa, 37°C, 10 min) as well as an untreated control were first single cell sorted into 96-well plates containing TSA, and incubated overnight.

To further study the physiological state of each sub-population, cultivability and heat sensitivity of each sub-population after HP treatment at 150 MPa, 37°C for 10 min was assessed. Spores were HP-treated, stained and gated as described in Section “Flow Cytometry Analysis”.

To assess cultivability, each sub-population was selected, and single cell sorted into three 96-well TSA plates as described above. Sorting started 25 min after decompression (including 16 min staining time). Experiments for each sub-population were repeated three times on different days independently (*n* = 3).

In order to investigate the heat resistance of each sub-population after HP treatment, each sub-population was sorted into 1.5 ml Eppendorf tubes using four-way purity precision (yield mask: 0; purity mask: 32; phase mask: 0). With this setting, the purity had the highest priority, and the counting accuracy was compromised to increase the sorting efficiency. The sorting block holding the Eppendorf tubes was connected to a water bath, and kept at 4°C. Sorted samples were directly used for further analysis when the percentage of this sub-population was large. For rare populations, the sorted samples were first diluted 10 times in order to obtain enough sample volume for further analysis. Samples were then plated on TSA plates with or without heating at 80°C for 10 min. A comparison of the number of survivals with and without heat treatment indicated whether the sorted sub-populations lost their heat resistance. Preliminary results showed that the staining did not influence the heat sensitivity of sorted cells at the chosen treatment conditions. Experiments were repeated three times on different days (*n* = 3) with technical duplicates. Results were expressed as mean value ± standard deviation.

## Results and Discussion

### Flow Cytometry as a Tool for High Pressure Spore Research

#### Heterogeneity and Dynamics of Spore Behavior Under HP

*B. subtilis* PS533 spores were HP-treated at 150 MPa and 37°C, stained with SYTO16 and PI, and analyzed using a flow cytometer. The optimized FCM staining and data collection protocol yielded reproducible results as verified with multiple technical replicates. The mean standard deviation of technical replicates within one sample was 0.3%, and the highest standard deviation observed was 1.3%. Compared to previous studies, the adapted protocol here with lower PI concentration and shorter PI incubation times yielded a better differentiation between PI-positive and PI-negative populations (Mathys et al., 2007; Reineke et al., 2013a).

**FIGURE 2:**
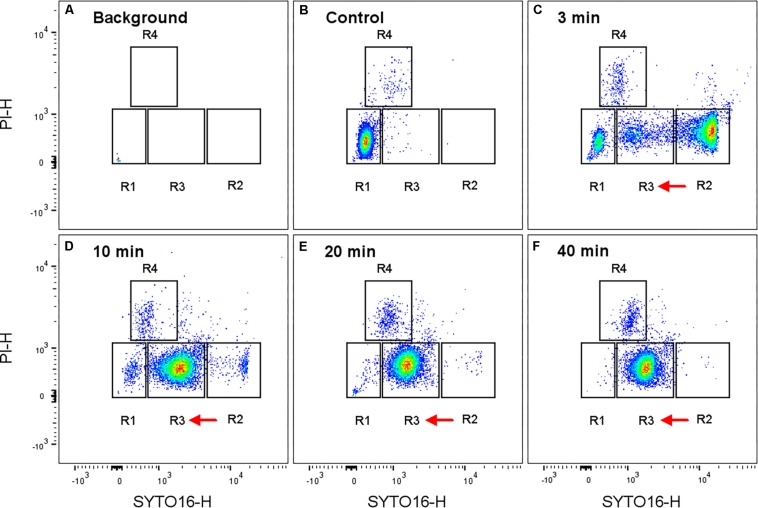
Representative flow cytometry plots for high pressure treated *Bacillus subtilis* spores at 150 MPa, 37°C. **(A)** Background signal (recorded for 30 sec to make it visible, with less than 4 events/s), **(B)** untreated, stained spores, and **(C–F)** were high pressure treated and stained spores. The high pressure treatment time was: **(C)** 3 min, **(D)** 10 min, **(E)** 20 min, and **(F)** 40 min. Events are depicted on a biexponential scale density plot of SYTO16 vs. PI revealing four different sub-populations R1- R4. The presumptive assignment of sub-populations is: R1: (super)dormant spores, R2: germinated spores, R3: previously referred to as “unknown”, R4: membrane-compromised spores. Red arrows indicate population shifts over the treatment time. Note that the recorded event rate for the background was less than 4 events/s compared to an average of 8000 recorded events/s for the samples. Hence, influence of background signal was negligible. Sub-population (R1-4) distribution percentages are given in Figure 3.

**FIGURE 3:**
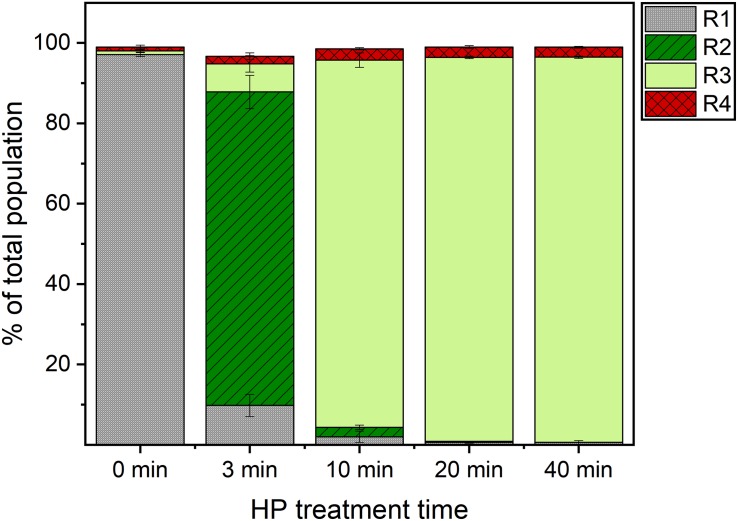
Sub-population distribution in flow cytometry analysis of high pressure treated *Bacillus subtilis* spores. Spores were treated in 50 mM ACES buffer (pH 7) at 150 MPa, 37°C for 3, 10, 20 and 40 min, stained with SYTO16 and PI, and analyzed in a flow cytometer. Four sub-populations were identified (R1–R4). The presumptive assignment is: R1: (super)dormant spores, R2: germinated spores, R3: previously referred to as “unknown”, R4: membrane-compromised spores. Error bars present standard deviations of three independent experiments (*n* = 3).

In line with results from a previous study on HP-treated *B. licheniformis* spores (Mathys et al., 2007), we also observed four distinctive sub-populations (R1-R4) of HP-treated *B. subtilis* spores (Figures 2, 3). Based on the assumption of stain permeability, these four sub-populations were previously suggested to include R1, a HPSD sub-population (SYTO16- and PI-negative); R2, a germinated sub-population (SYTO16-positive and PI-negative); R3, an “unknown” sub-population whose physiological state was unknown (intermediate SYTO16 signal, PI-negative), and R4, an inactivated sub-population (PI-positive) (Mathys et al., 2007; Reineke et al., 2013a). Occasionally, two sub-populations seem to appear in gate R1. The reason for this occasional appearance is unclear. In our case, these sub-populations were gated as one single HPSD sub-population. The main reason for this approach was that the gate for R1 was drawn according to a stained control of dormant spores.

The majority of spores ended up in the R2 domain (i.e., presumably germinated domain) already after 3 min of HP treatment. A population shift from the R2 to the unknown R3 domain occurred with increasing HP treatment time (Figures 2, 3). While the percentages of the four sub-populations varied between spore batches, this kinetic behavior under HP was observed for every batch. This finding therefore clearly suggests that the unknown domain (R3) after 10 min of HP treatment included already germinated spores rather than spores on their way to germination. This is also in line with Mathys et al. (2007) suggesting a three-step spore inactivation model at 150 MPa involving a germination step, followed by an unknown step, and a final inactivation step.

#### Dynamic Germination Behavior After Decompression

As demonstrated above, spore germination behavior under HP was dynamic. Therefore, it was of interest to understand whether these dynamics will stop after decompression or whether changes of their physiological states will continue. The influence of time between HP treatment and FCM analysis was investigated in order to take potential changes in sub-population distribution after decompression into account. Treated samples were analyzed at 25, 90, 150, and 180 min after decompression. Time between decompression and FCM analysis exerted no effect on the distribution of different sub-populations after 10 min HP treatment. For the 3 min treatment, however, sub-population distribution was shifted within 25 and 90 min after decompression. While 68 ± 3% of spores were found within the presumably germinated domain (R2) at 25 min following decompression, this ratio increased to 84 ± 2% at 90 min after decompression. The sub-population distribution mostly remained stable after 90 min. This finding suggests that even after removing the HP as a germination trigger, germination interestingly proceeded. This is similar to the so-called “commitment” concept that has been described for nutrient germination, where spores become committed to germination as soon as they encounter nutrients, and germination proceeds even after removal of the germinant (Setlow, 2003). Previous findings by Kong et al. (2014) also strongly suggest that this concept applies to HP germination at 150 MPa. As the process of HP germination at 150 MPa is believed to be similar to the process of nutrient germination, this observation fits well into current knowledge on germination via germinant receptors (Wuytack et al., 1998; Paidhungat et al., 2002; Black et al., 2005). The concept of commitment is an important aspect to be considered in industrial applications. Therefore, timing of a possible subsequent mild inactivation step needs to be well-adjusted. On one hand, subsequent inactivation should start only upon loss of resistance. On the other hand, the time between germination and inactivation should be minimized to avoid outgrowth and potential toxin production.

#### Comparison of Results From FCM and Classical Methods to Study Spore Germination in the Overall Population

Flow cytometry results were compared quantitatively with those obtained using the viable plate count method for a 10 min HP treatment at 150 MPa and 37°C. The HPSD spore ratios from viable plate count method and FCM were 0.6 ± 0.2 and 1.6 ± 0.5%, respectively. Both methods thus yielded very similar results in terms of HPSD fraction. FCM provided an advantage of speed, as results were generated within 1 h, whereas plating results were only available the next day. However, the ratios of presumably-inactivated spores determined by FCM (R4) and the viable plate count method deviated by up to 58%. Plate count and FCM yielded 45 ± 14 and 2.7 ± 0.1% inactivated cells, respectively. This deviation may be partly explained by the fact that the numbers from these two different methods do not actually represent exactly the same physiological state. The number from FCM analysis represents spores with compromised cell membranes emitting a PI-positive signal. This number does not include cells that still have an intact membrane but are actually not culturable anymore, i.e., viable but non-culturable bacteria with intact membranes. On the other hand, the number obtained from the plate count reflects all non-culturable cells regardless of cell membrane integrity (Nebe-von-Caron et al., 2000; Oliver, 2016). Some germinated spores may have been sub-lethally damaged during HP treatment, and maintained intact membranes (i.e., did not take up PI), however, might have difficulties to be recovered on the chosen media. Single cell sorting revealed that some cells in R3 were also unable to grow on nutrient-rich media (see section “Physiological State of Each Sub-population After HP Treatment” below). This might explain the deviation between ratios of presumably-inactivated (R4) spores obtained from the two methods.

**FIGURE 4:**
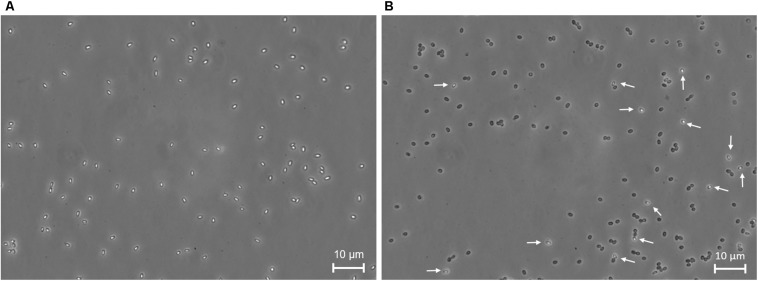
Phase-contrast microscopy images of *Bacillus subtilis* spores. **(A)** Untreated and **(B)** 3 min high pressure treated spores at 150 MPa and 37°C. White arrows indicate phase-bright spores, i.e., spores that remained dormant after high pressure treatment.

Flow cytometry results were also compared qualitatively with those from phase-contrast microscopic analysis. Phase-contrast microscopic analysis clearly showed that most treated spores turned phase-dark already after 3 min of HP treatment (Figure 4). This indicates that germination already took place at this point, which is in line with our finding that the so far unknown sub-population (R3) already germinated. Phase-contrast microscopy allows a quick and rough estimation of germination, yet quantifying the percentage of phase-bright spores for more reliable results with this method is laborious. To this end, FCM is more advantageous, as it allows analysis of 15000 events in less than 2 s.

While the developed FCM protocol quickly provides information on structural properties of spore sub-populations (e.g., presence/absence of cortex and membrane integrity), no information regarding recoverability of the treated spores is obtained. In food applications, however, information regarding the physiological fitness and sensitivity of spores after the treatment is of paramount importance for accurate assessment of the safety of a preservation strategy, and prevention of the outgrowth of sub-lethally damaged cells. The plating of the overall population provided some further insights, yet no understanding of the physiological fitness of individual sub-populations. Sub-populations were therefore sorted, and further analyzed to assess their physiological state. Before further characterization via sorting, however, the influence of staining on the cultivability of sorted cells was investigated.

### Influence of Staining on Cultivability of Sorted Cells

**FIGURE 5:**
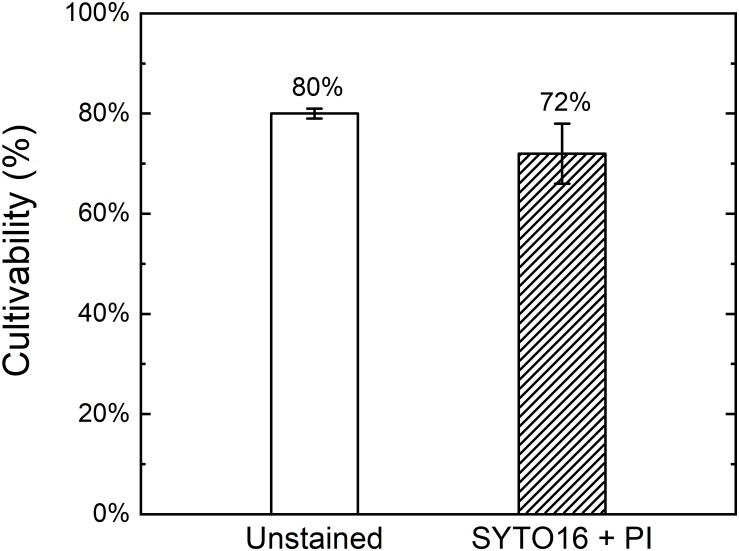
Cultivability of stained and unstained high pressure treated *Bacillus subtilis* spores. Spores were high pressure treated at 150 MPa and 37°C for 10 min in 50 mM ACES buffer at pH 7, left unstained or stained with SYTO16 and PI, and single cell sorted into a 96-well plate containing tryptic soy agar through FACS. Error bars present standard deviations (*n* = 3).

The cultivabilities of stained and unstained HP-treated spores (150 MPa, 37°C, 10 min) were compared with each other to investigate the effect of staining. Average cultivability of unstained HP-treated spores was around 80%, whereas that of the same sample stained with SYTO16 and PI was around 72% (Figure 5). The cultivability thus decreased by around 8% upon staining. Hence, staining only marginally influenced the cultivability of the treated sample.

### Physiological State of Each Sub-Population After HP Treatment

**FIGURE 6:**
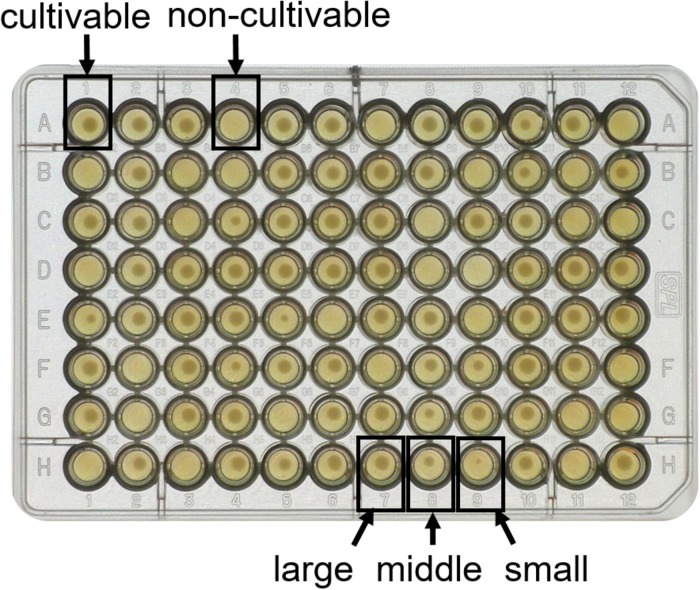
Heterogeneous colony growth of FACS single cell sorted high pressure treated *Bacillus subtilis* spores. Spores were high pressure treated at 150 MPa and 37°C for 10 min in 50 mM ACES buffer at pH 7, and single cells were sorted into a 96-well plate containing tryptic soy agar using FACS. Some wells displayed no growth, e.g., well A4 (non-cultivable), while other wells showed growth, e.g., well A1 (cultivable). Among the cultivable cells, large differences in colony size were observed, e.g., well H7 (large), H8 (middle) and H9 (small).

In order to get an impression of the effect of HP on the physiological state of spores, treated and untreated samples were single cell sorted into 96-well agar plates, and incubated overnight to allow colony growth. Colony growth of treated spores was very heterogeneous compared to the untreated control. In the untreated control, colony growth was observed in 97 ± 0.4% of wells, and sizes of all colonies were approximately the same. In contrast, wells of the treated sample showed either no colony growth or small, middle or large-sized colonies (Figure 6). Our hypothesis is that some spores may have been sub-lethally damaged during HP treatment, and needed time to repair the damages before proliferating to form a visible colony. These sub-lethally damaged cells then appeared as smaller colonies.

**FIGURE 7:**
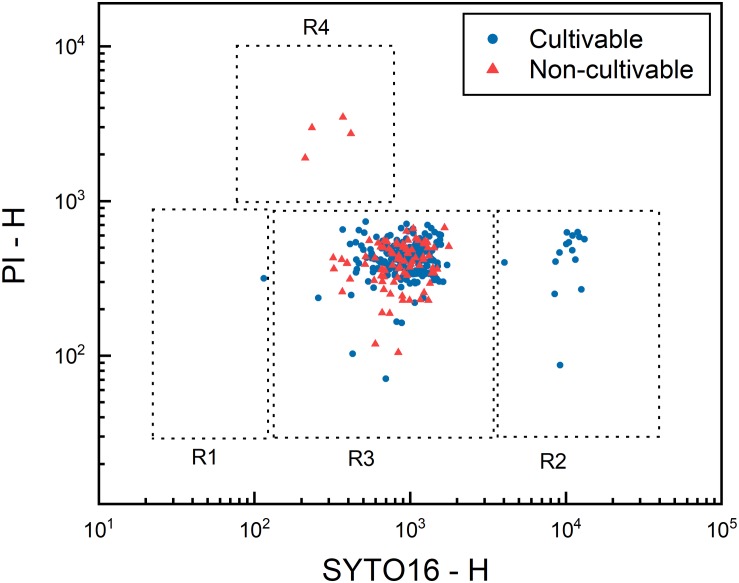
Distribution of cultivability of high pressure treated *Bacillus subtilis* spores. Spores were high pressure treated at 150 MPa and 37°C for 10 min in 50 mM ACES buffer at pH 7, stained with SYTO16 and PI, and single cell sorted into 96-well plates filled with tryptic soy agar through FACS. After 48 h of incubation, colony formation in three 96-well plates, in total 288 wells, was recorded as “cultivable” or “non-cultivable” depending on the presence or absence of a colony in each well. SYTO16 and PI signals recorded during the sorting using the index sorting function are plotted to visualize the distribution of cultivability. Blue dots and red triangles represent cultivable and non-cultivable events, respectively. Dashed lines indicate approximate gate locations of the four presumptive sub-populations.

Data collected using the index sorting function clearly indicated that most non-cultivable events emitted either high PI signal (R4), which are presumably inactivated spores, or intermediate SYTO16 signal (R3), of which the physiological state was previously referred to as “unknown” (Figure 7).

**FIGURE 8:**
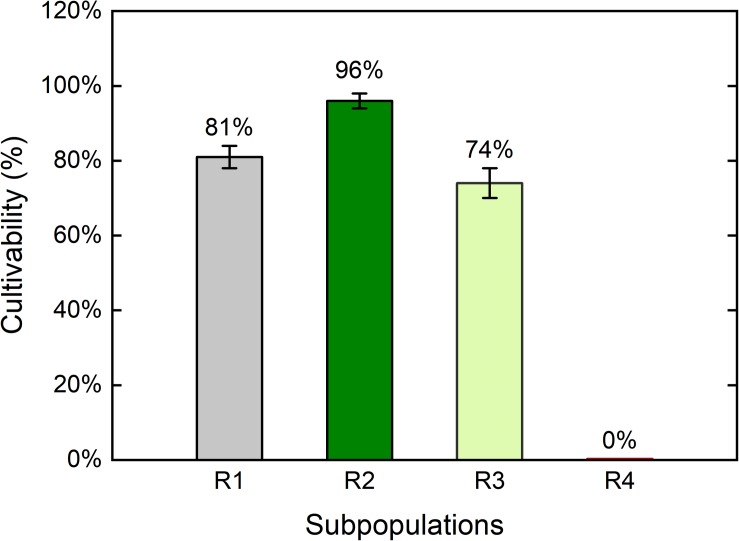
Cultivability of the four *Bacillus subtilis* sub-populations after high pressure treatment. Spores were treated at 150 MPa and 37°C for 10 min in 50 mM ACES buffer at pH 7, stained with SYTO16 and PI. Four sub-populations were observed with the following presumptive assignment: R1: superdormant spores, R2: germinated spores, R3: previously referred to as “unknown”, R4: membrane-compromised spores. Each sub-population was single cell sorted into 96-well plates containing tryptic soy agar through FACS and incubated at 37°C for 48 h. Error bars present standard deviations of three independent treatments (*n* = 3).

Cultivability of each sub-population after HP treatment (150 MPa, 37°C, 10 min) was further characterized by single cell sorting of each sub-population into 96-well TSA plates (Figure 8). The presumably germinated sub-population (R2) yielded the highest cultivability at approximately 96%, indicating that almost all spores in this sub-population were cultivable. Cultivability of the presumably HPSD sub-population (R1) was around 81%. The lower cultivability of the HPSD sub-population than that of the germinated sub-population may be explained by some HPSD spores being also nutrient superdormant and unable to germinate on TSA agar. The sub-population R4 (presumably-inactivated) yielded 0% cultivability. This strongly indicates that these spores were indeed inactivated by HP treatment. However, it is also possible that these spores survived the HP treatment, yet became very vulnerable and could not germinate due to additional stresses induced by staining and sorting procedures.

Importantly, we demonstrated here for the first time the cultivability of the unknown sub-population (R3) with direct evidence. Previous studies on HP-treated *B. subtilis* and *B. licheniformis* spores also identified one or two sub-populations with intermediate SYTO16 signals, which were referred to as “unknown” sub-population due to unclear physiological state. The cultivability of these sub-populations were proposed by comparing the FCM results with plate count data on the overall population. No previous study isolated and cultivated the single sub-populations (Mathys et al., 2007; Reineke et al., 2012; Borch-Pedersen et al., 2017). For example, some researchers found one sub-population with intermediate SYTO16 signal, and suggested that cells in this sub-population were not cultivable (Mathys et al., 2007; Reineke et al., 2012). Other researchers found two “unknown” sub-populations, and suggested one of them to be viable and the other non-viable (Borch-Pedersen et al., 2017). Here, the cultivability of the previously unknown sub-population (R3) was around 74%. Hence, even though cells with intermediate SYTO16 intensity formed one sub-population in FCM analysis, it included cells with different fitness levels. The cultivability of the sub-population R3 was around 22% lower than that of the germinated sub-population (R2). Unlike the sorted cells in the R2 sub-population with homogenous colony sizes, the sorted R3 sub-population included cells with very different colony sizes as in Figure 6. This heterogeneous growth may imply that certain cells were sub-lethally damaged, or at least less fit than others.

**FIGURE 9:**
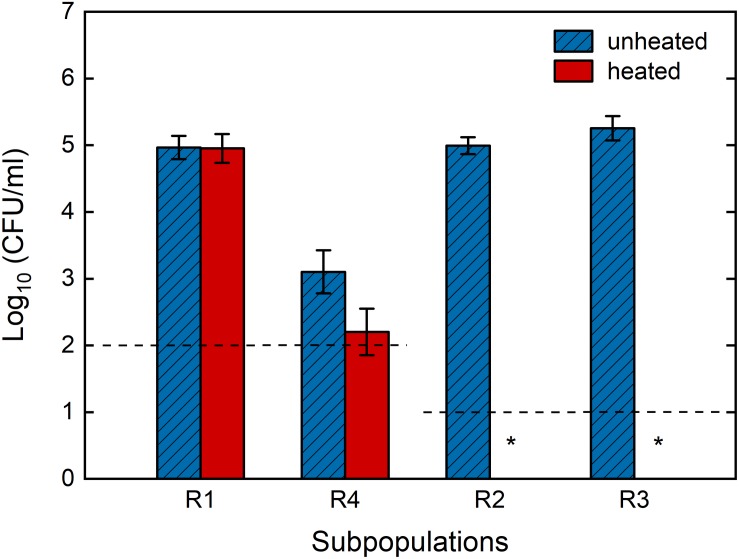
Survival of individual *Bacillus subtilis* sub-populations after high pressure treatment with and without additional heat treatment. Spores were high pressure treated in 50 mM ACES buffer (pH 7) at 150 MPa and 37°C for 10 min, stained with SYTO16 and PI, and each sub-population was sorted with FACS and plated on TSA plates with and without heat treatment at 80°C for 10 min in order to assess loss of heat resistance. Dashed lines indicate the detection limit and ^∗^ indicate no colonies were detected after 48 h incubation. Sorted R1 and R4 groups were first diluted approximately 10 times in order to obtain enough sample volume for the analysis, which lead to a higher detection limit compared to R2 and R3. The presumptive sub-population assignment is R1: superdormant spores, R2: germinated spores, R3: previously referred to as “unknown”, R4: membrane-compromised spores. Error bars present standard deviations of three independent analyses (*n* = 3).

In addition to cultivability, we also characterized the heat resistance of each sub-population. The four sub-populations were sorted into four 1.5 ml Eppendorf tubes, and plated on TSA plates with or without heat treatment at 80°C for 10 min. A comparison between the CFU numbers from heated and unheated samples provided information regarding the heat resistance loss of each sub-population (Figure 9). As expected, cells in the R1 sub-population, which were presumably HPSD spores, maintained their heat resistance. This finding confirms that this sub-population indeed included spores that remained dormant after the HP treatment. None of the cells sorted from the previously unknown sub-population (R3) survived heat treatment, indicating complete loss of heat resistance. The same observation applies for the sub-population R2. The loss of heat resistance in sub-populations R2 and R3 clearly suggests that both sub-populations germinated through HP treatment, in line with our findings on kinetic transition behavior in Section “Heterogeneity and Dynamics of Spore Behavior Under HP.” Rather unexpectedly, some spores that fell into the presumably-inactivated sub-population (R4) were actually cultivable and a few of the cultivable cells did not lose their heat resistance. This is not in line with the single cell sorting on 96-well plates, where none of the spores that ended up in the gating window of R4 were cultivable. In this case, however, only 1% of the R4 group could grow and among those 90% lost its heat resistance.

The results of the investigation on the physiological states of the four sub-populations confirmed that the assigned sub-populations based on dye permeability mostly reproduced the expected physiological states: the R1 sub-population was confirmed to include HPSD spores, which remained heat-resistant and could mostly germinate and grow on rich medium. The R4 sub-population included mostly lethally-damaged spores that could not be recovered on rich medium. The R2 sub-population consisted of mostly cultivable cells with lost heat resistance, indicating that they germinated. Moreover, it is now clear that the spores that ended up in the previously unknown R3 gating window were clearly heat-sensitive and partially-cultivable germinated spores.

## Conclusion

In this study, we optimized and thoroughly validated the use of FCM and FACS in combination with SYTO16- and PI-staining for studying *Bacillus* spore behavior under HP. This work provides an optimized, comprehensive and detailed protocol for analysis of HP treated spores with FCM. Four sub-populations were detected and isolated using FACS. Properties of these sub-populations, including heat resistance and cultivability, were characterized. The four sub-populations included (1) heat resistant and mostly cultivable HPSD spores (PI- and SYTO16-negative), (2) heat sensitive and cultivable germinated spores (SYTO16-positive and PI-negative), (3) heat sensitive germinated spores with compromised cultivability (intermediate SYTO16 signal, PI negative) and (4) membrane-compromised cells with barely detectable cultivability (PI-positive). Previous studies referred to the sub-population with high SYTO16 intensity as “germinated,” whereas spores with intermediate SYTO16 intensity were referred to as “unknown” sub-population since the physiological state of this sub-population was poorly understood. Based on our findings, we suggest renaming sub-populations with high and intermediate SYTO16 intensities as “germinated with high physiological fitness” and “germinated with partial sublethal damage,” respectively. For future research, it would be of interest to study this potential sub-lethal damage in greater detail, e.g., by looking for decreased plating efficiencies in unfavorable conditions (Ray, 1979; Somolinos et al., 2008; Schottroff et al., 2018; Lv et al., 2019). Further, it would be of interest to get a better understanding of the structural nature of this sublethal damage.

Kinetic transition findings further suggested the following transition of spores at 150 MPa and 37°C: dormant → germinated with high physiological fitness → germinated with partial sublethal damage → membrane-compromised, i.e., presumably inactivated. Most spores germinated within a few minutes of HP treatment at 150 MPa and 37°C, yet only few spores were completely inactivated. Instead, most spores ended up in a germinated and partially sub-lethally damaged state after 10 min treatment. These cells were still partially able to grow in nutrient-rich environments. Application of a subsequent mild follow-up treatment, such as mild heating, is therefore crucial for complete inactivation of spores. When thinking about follow-up treatments, timing is a crucial factor. Here, we showed that germination continued even after HP treatment. Therefore, it is important to apply the follow up treatment only after germination and loss of resistance. On the other hand, excessive waiting times between HP and follow up treatments may allow some of the germinated spores to proliferate, and potentially produce heat stable toxins. Therefore, timing in germination-inactivation strategies needs to be well-adjusted, and further investigations into this aspect are needed.

While FCM and FACS proved to be perfectly suitable for studying heterogeneous germination behavior of *B. subtilis* spores under HP conditions at 150 MPa and 37°C, these methods may not be as suitable to study other *Bacillus* species or use with other pressure/temperature ranges. Therefore, suitability of these methods for chosen strains and treatment conditions must be thoroughly evaluated, and methods should be adapted when necessary. Once suitability is confirmed, FCM offers great potential for rapid and quantitative analysis of heterogeneity within a treated sample with high statistical power. The use of FACS offers even more opportunities, e.g., when combined with molecular techniques like (single cell) sequencing, for elucidating mechanisms and underlying genetic roots of heterogeneous spore behavior. A highly-relevant and important next step would be the isolation and detailed characterization of the HPSD fraction, as this is a major hurdle for a successful industrial application of a mild germination-inactivation strategy.

In summary, we provided a solid foundation for future research on heterogeneous spore behavior under HP. Our findings will improve our understanding of heterogeneity in spore germination, and assist further development of HP-based spore control technologies.

## Data Availability Statement

The datasets generated for this study are available on request to the corresponding author.

## Author Contributions

YZ, AD, CO, SB, and AM contributed to all stages of the work. YZ, AD, and AM conceived the idea, designed and conducted experiments, and wrote the manuscript. CO contributed mainly to setup and conduction of experiments. SB contributed mainly to the design, setup and interpretation of FCM experiments.

## Conflict of Interest

The authors declare that the research was conducted in the absence of any commercial or financial relationships that could be construed as a potential conflict of interest.

## References

[B1] Abee T. Groot M. N. Tempelaars M. Zwietering M. Moezelaar R. van der Voort M. (2011). Germination and outgrowth of spores of *Bacillus cereus* group members: diversity and role of germinant receptors. *Food Microbiol.* 28 199–208. 10.1016/j.fm.2010.03.015 21315974

[B2] Alvarez D. F. Helm K. Degregori J. Roederer M. Majka S. (2010). Publishing flow cytometry data. *Am. J. Physiol. Lung Cell Mol. Physiol.* 298 L127–L130. 10.1152/ajplung.00313.2009 19915158 PMC2822558

[B3] Ambriz-Avina V. Contreras-Garduno J. A. Pedraza-Reyes M. (2014). Applications of flow cytometry to characterize bacterial physiological responses. *Biomed. Res. Int.* 2014:461941. 10.1155/2014/461941 25276788 PMC4174974

[B4] Baier D. Reineke K. Doehner I. Mathys A. Knorr D. (2011). Fluorescence-based methods for the detection of pressure-induced spore germination and inactivation. *High Press. Res.* 31 110–115. 10.1080/08957959.2010.527338

[B5] Black E. P. Koziol-Dube K. Guan D. S. Wei H. Setlow B. Cortezzo D. E. (2005). Factors influencing germination of *Bacillus subtilis* spores via activation of nutrient receptors by high pressure. *Appl. Environ. Microbiol.* 71 5879–5887. 10.1128/Aem.71.10.5879-5887.2005 16204500 PMC1265928

[B6] Black E. P. Wei J. Atluri S. Cortezzo D. E. Koziol-Dube K. Hoover D. G. (2007). Analysis of factors influencing the rate of germination of spores of *Bacillus subtilis* by very high pressure. *J. Appl. Microbiol.* 102 65–76. 10.1111/j.1365-2672.2006.03062.x 17184321

[B7] Borch-Pedersen K. Mellegard H. Reineke K. Boysen P. Sevenich R. Lindback T. (2017). Effects of high pressure on *Bacillus licheniformis* spore germination and inactivation. *Appl. Environ. Microbiol.* 83 e2108–e2117. 10.1128/AEM.00503-17 28476768 PMC5494625

[B8] Chen D. Huang S. S. Li Y. Q. (2006). Real-time detection of kinetic germination and heterogeneity of single *Bacillus* spores by laser tweezers Raman spectroscopy. *Anal. Chem.* 78 6936–6941. 10.1021/ac061090e 17007517

[B9] Collado J. Fernandez A. Rodrigo M. Martinez A. (2004). Variation of the spore population of a natural source strain of *Bacillus cereus* in the presence of inosine. *J. Food. Prot.* 67 934–938. 10.4315/0362-028X-67.5.934 15151230

[B10] Dong P. Georget E. S. Aganovic K. Heinz V. Mathys A. (2015). Ultra high pressure homogenization (UHPH) inactivation of *Bacillus amyloliquefaciens* spores in phosphate buffered saline (PBS) and milk. *Front. Microbiol.* 6:712. 10.3389/fmicb.2015.00712 26236296 PMC4500962

[B11] Doona C. J. Feeherry F. E. Ross E. W. Kustin K. (2016). Chemical kinetics for the microbial safety of foods treated with high pressure processing or hurdles. *Food Eng. Rev.* 8 272–291. 10.1007/s12393-015-9138-7

[B12] Doona C. J. Ghosh S. Feeherry F. F. Ramirez-Peralta A. Huang Y. Chen H. (2014). High pressure germination of *Bacillus subtilis* spores with alterations in levels and types of germination proteins. *J. Appl. Microbiol.* 117 711–720. 10.1111/jam.12557 24891141

[B13] Georget E. Kapoor S. Winter R. Reineke K. Song Y. Y. Callanan M. (2014a). In situ investigation of *Geobacillus stearothermophilus* spore germination and inactivation mechanisms under moderate high pressure. *Food Microbiol.* 41 8–18. 10.1016/j.fm.2014.01.007 24750808

[B14] Georget E. Miller B. Callanan M. Heinz V. Mathys A. (2014b). (Ultra) high pressure homogenization for continuous high pressure sterilization of pumpable foods - a review. *Front. Nutr.* 1:15. 10.3389/fnut.2014.00015 25988118 PMC4428391

[B15] Georget E. Kushman A. Callanan M. Ananta E. Heinz V. Mathys A. (2014). *Geobacillus stearothermophilus* ATCC 7953 spores chemical germination mechanisms in model systems. *Food Control* 50 141–149. 10.1016/j.foodcont.2014.08.044

[B16] Georget E. Sauvageat J. L. Burbidge A. Mathys A. (2013). Residence time distributions in a modular micro reaction system. *J. Food Eng.* 116 910–919. 10.1016/j.jfoodeng.2013.01.041

[B17] Ghosh S. Setlow P. (2009). Isolation and characterization of superdormant spores of *Bacillus* species. *J. Bacteriol.* 191 1787–1797. 10.1128/Jb.01668-08 19136594 PMC2648361

[B18] Gould G. W. (1970). Symposium on bacterial spores: IV. Germination and the problem of dormancy. *J. Appl. Bacteriol.* 33 34–49. 10.1111/j.1365-2672.1970.tb05232.x 4246071

[B19] Gould G. W. (2006). History of science - spores. *J. Appl. Microbiol.* 101 507–513. 10.1111/j.1365-2672.2006.02888.x 16907801

[B20] Gould G. W. Sale A. J. H. (1970). Initiation of germination of bacterial spores by hydrostatic pressure. *J. Gen. Microbiol.* 60:335. 10.1099/00221287-60-3-335 5487618

[B21] Hashimoto T. Frieben W. R. Conti S. F. (1969). Germination of single bacterial spores. *J. Bacteriol.* 98 1011–1020. 4977979 10.1128/jb.98.3.1011-1020.1969 PMC315288

[B22] Karava M. Bracharz F. Kabisch J. (2019). Quantification and isolation of *Bacillus subtilis* spores using cell sorting and automated gating. *PLoS One* 14:e0219892. 10.1371/journal.pone.0219892 31356641 PMC6663000

[B23] Knorr D. Heinz H. Schlüter O. Zenker M. (1998). “The potential impact of high pressure as unit operation for food processing,” in *High Pressure Food Science, Bioscience and Chemistry*, ed. Isaacs N. S. (Cambridge: The Royal Society of Chemistry), 227–235. 10.1533/9781845698379.3.227

[B24] Knorr D. Reineke K. Mathys A. Heinz V. Buckow R. (2010). “High-pressure-induced effects on bacterial spores, vegetative microorganisms, and enzymes,” in *Food Engineering Interfaces*, eds Aguilera J. M. Simpson R. Welti-Chanes J. Bermudez-Aguirre D. Barbosa-Canovas G. (New York, NY: Springer), 325–340. 10.1007/978-1-4419-7475-4_14

[B25] Kong L. Doona C. J. Setlow P. Li Y. Q. (2014). Monitoring rates and heterogeneity of high-pressure germination of *Bacillus* spores by phase-contrast microscopy of individual spores. *Appl. Environ. Microbiol.* 80 345–353. 10.1128/AEM.03043-13 24162576 PMC3911021

[B26] Kong L. Zhang P. Yu J. Setlow P. Li Y. Q. (2010). Monitoring the kinetics of uptake of a nucleic acid dye during the germination of single spores of *Bacillus* species. *Anal. Chem.* 82 8717–8724. 10.1021/ac1022327 20873796

[B27] Kong L. B. Zhang P. F. Wang G. W. Yu J. Setlow P. Li Y. Q. (2011). Characterization of bacterial spore germination using phase-contrast and fluorescence microscopy, Raman spectroscopy and optical tweezers. *Nat. Protoc.* 6 625–639. 10.1038/nprot.2011.307 21527920

[B28] Lovdal I. S. Hovda M. B. Granum P. E. Rosnes J. T. (2011). Promoting *Bacillus cereus* spore germination for subsequent inactivation by mild heat treatment. *J. Food. Prot.* 74 2079–2089. 10.4315/0362-028x.Jfp-11-292 22186048

[B29] Luu S. Cruz-Mora J. Setlow B. Feeherry F. E. Doona C. J. Setlow P. (2015). The effects of heat activation on *Bacillus* spore germination, with nutrients or under high pressure, with or without various germination proteins. *Appl. Environ. Microbiol.* 81 2927–2938. 10.1128/AEM.00193-15 25681191 PMC4375313

[B30] Lv R. L. Wang D. L. Zou M. M. Wang W. J. Ma X. B. Chen W. J. (2019). Analysis of *Bacillus cereus* cell viability, sublethal injury, and death induced by mild thermal treatment. *J. Food Saf.* 39:e12581.

[B31] Margosch D. Ehrmann M. A. Buckow R. Heinz V. Vogel R. F. Ganzle M. G. (2006). High-pressure-mediated survival of *Clostridium botulinum* and *Bacillus amyloliquefaciens* endospores at high temperature. *Appl. Environ. Microbiol.* 72 3476–3481. 10.1128/Aem.72.5.3476-3481.2006 16672493 PMC1472378

[B32] Margosch D. Gaenzle M. G. Ehrmann M. A. Vogel R. F. (2004). Pressure inactivation of *Bacillus* endospores. *Appl. Environ. Microbiol.* 70 7321–7328. 10.1128/aem.70.12.7321-7328.2004 15574932 PMC535133

[B33] Martinez-Monteagudo S. I. Ganzle M. G. Saldana M. D. A. (2014). High-pressure and temperature effects on the inactivation of *Bacillus amyloliquefaciens*, alkaline phosphatase and storage stability of conjugated linoleic acid in milk. *Innov. Food Sci. Emerg. Technol.* 26 59–66. 10.1016/j.ifset.2014.05.003

[B34] Mathys A. Chapman B. Bull M. Heinz V. Knorr D. (2007). Flow cytometric assessment of *Bacillus* spore response to high pressure and heat. *Innov. Food Sci. Emerg. Technol.* 8 519–527. 10.1016/j.ifset.2007.06.010

[B35] Nebe-von-Caron G. Stephens P. J. Hewitt C. J. Powell J. R. Badley R. A. (2000). Analysis of bacterial function by multi-colour fluorescence flow cytometry and single cell sorting. *J. Microbiol. Methods* 42 97–114. 10.1016/S0167-7012(00)00181-0 11000436

[B36] Nicholson W. L. Setlow P. (1990). “Sporulation, germination and outgrowth,” in *Molecular biological methods for Bacillus*, eds Harwood C. R. Chambert R. Cutting S. M. (Chichester: Wiley), 391–450.

[B37] Oliver J. D. (2016). The viable but nonculturable state for bacteria: status update. *Microbe* 11 159–164. 10.1128/microbe.11.159.1

[B38] Paidhungat M. Setlow B. Daniels W. B. Hoover D. Papafragkou E. Setlow P. (2002). Mechanisms of induction of germination of *Bacillus subtilis* spores by high pressure. *Appl. Environ. Microbiol.* 68 3172–3175. 10.1128/aem.68.6.3172-3175.2002 12039788 PMC123951

[B39] Pandey R. Ter Beek A. Vischer N. O. Smelt J. P. Brul S. Manders E. M. (2013). Live cell imaging of germination and outgrowth of individual *Bacillus subtilis* spores; the effect of heat stress quantitatively analyzed with SporeTracker. *PLoS One* 8:e58972. 10.1371/journal.pone.0058972 23536843 PMC3607599

[B40] Parks D. R. Roederer M. Moore W. A. (2006). A new “Logicle” display method avoids deceptive effects of logarithmic scaling for low signals and compensated data. *Cytometry A* 69a 541–551. 10.1002/cyto.a.20258 16604519

[B41] Patrignani F. Lanciotti R. (2016). Applications of high and ultra high pressure homogenization for food safety. *Front. Microbiol.* 7:1132. 10.3389/fmicb.2016.01132 27536270 PMC4971028

[B42] Ragkousi K. Cowan A. E. Ross M. A. Setlow P. (2000). Analysis of nucleoid morphology during germination and outgrowth of spores of *Bacillus* species. *J. Bacteriol.* 182 5556–5562. 10.1128/jb.182.19.5556-5562.2000 10986261 PMC111001

[B43] Ray B. (1979). Methods to detect stressed microorganisms. *J. Food Protect.* 42 346–355. 10.4315/0362-028x-42.4.346 30812186

[B44] Reineke K. Doehner I. Schlumbach K. Baier D. Mathys A. Knorr D. (2012). The different pathways of spore germination and inactivation in dependence of pressure and temperature. *Innov. Food Sci. Emerg. Technol.* 13 31–41. 10.1016/j.ifset.2011.09.006

[B45] Reineke K. Ellinger N. Berger D. Baier D. Mathys A. Setlow P. (2013a). Structural analysis of high pressure treated *Bacillus subtilis* spores. *Innov. Food Sci. Emerg. Technol.* 17 43–53. 10.1016/j.ifset.2012.10.009 10376818

[B46] Reineke K. Mathys A. Heinz V. Knorr D. (2013b). Mechanisms of endospore inactivation under high pressure. *Trends Microbiol.* 21 296–304. 10.1016/j.tim.2013.03.001 23540831

[B47] Reineke K. Mathys A. (2020). Endospore inactivation by emerging technologies: a review of target structures and inactivation mechanisms. *Annu. Rev. Food Sci. Technol.* 11:e051632. 10.1146/annurev-food-032519-051632 31905011

[B48] Reineke K. Mathys A. Knorr D. (2011). The impact of high pressure and temperature on bacterial spores: inactivation mechanisms of *Bacillus subtilis* above 500 MPa. *J. Food Sci.* 76 M189–M197. 10.1111/j.1750-3841.2011.02066.x 21535843

[B49] Schottroff F. Frohling A. Zunabovic-Pichler M. Krottenthaler A. Schluter O. Jager H. (2018). Sublethal injury and viable but non-culturable (VBNC) state in microorganisms during preservation of food and biological materials by non-thermal processes. *Front. Microbiol.* 9:e02773. 10.3389/fmicb.2018.02773 30515140 PMC6255932

[B50] Setlow B. Setlow P. (1996). Role of DNA repair in *Bacillus subtilis* spore resistance. *J. Bacteriol.* 178 3486–3495. 10.1128/jb.178.12.3486-3495.1996 8655545 PMC178117

[B51] Setlow P. (2003). Spore germination. *Curr. Opin. Microbiol.* 6 550–556. 10.1016/j.mib.2003.10.001 14662349

[B52] Setlow P. (2006). Spores of *Bacillus subtilis*: their resistance to and killing by radiation, heat and chemicals. *J. Appl. Microbiol.* 101 514–525. 10.1111/j.1365-2672.2005.02736.x 16907802

[B53] Setlow P. (2007). I will survive: DNA protection in bacterial spores. *Trends Microbiol.* 15 172–180. 10.1016/j.tim.2007.02.004 17336071

[B54] Setlow P. (2014). Germination of spores of *Bacillus* species: what we know and do not know. *J. Bacteriol.* 196 1297–1305. 10.1128/JB.01455-13 24488313 PMC3993344

[B55] Setlow P. Johnson E. A. (2007). “Spores and their significance,” in *Food Microbiology: Fundamentals and Frontiers*, 3rd Edn, eds Doyle M. Buchanan R. (Washington, DC: ASM Press), 35–67. 10.1128/9781555815912.ch3

[B56] Setlow P. Wang S. W. Li Y. Q. (2017). Germination of spores of the orders Bacillales and Clostridiales. *Annu. Rev. Microbiol.* 71 459–477. 10.1146/annurev-micro-090816-093558 28697670

[B57] Sevenich R. Mathys A. (2018). Continuous versus discontinuous ultra-high-pressure systems for food sterilization with focus on ultra-high-pressure homogenization and high-pressure thermal sterilization: a review. *Compr. Rev. Food Sci. Food Saf.* 17 646–662. 10.1111/1541-4337.12348 33350130

[B58] Somolinos M. Garcia D. Pagan R. Mackey B. (2008). Relationship between sublethal injury and microbial inactivation by the combination of high hydrostatic pressure and citral or tert-butyl hydroquinone. *Appl. Environ. Microbiol.* 74 7570–7577. 10.1128/Aem.00936-08 18952869 PMC2607182

[B59] Stiefel P. Schmidt-Emrich S. Maniura-Weber K. Ren Q. (2015). Critical aspects of using bacterial cell viability assays with the fluorophores SYTO9 and propidium iodide. *BMC Microbiol.* 15:36. 10.1186/s12866-015-0376-x 25881030 PMC4337318

[B60] Storz G. Hengge R. (2010). *Bacterial Stress Responses.* Washington, DC: ASM Press.

[B61] Trunet C. Carlin F. Coroller L. (2017). Investigating germination and outgrowth of bacterial spores at several scales. *Trends Food Sci. Technol.* 64 60–68. 10.1016/j.tifs.2017.03.008

[B62] Ueckert J. Breeuwer P. Abee T. Stephens P. von Caron G. N. ter Steeg P. F. (1995). Flow cytometry applications in physiological study and detection of foodborne microorganisms. *Int. J. Food Microbiol.* 28 317–326. 10.1016/0168-1605(95)00066-6 8750676

[B63] van Melis C. C. den Besten H. M. Nierop Groot M. N. Abee T. (2014). Quantification of the impact of single and multiple mild stresses on outgrowth heterogeneity of *Bacillus cereus* spores. *Int. J. Food Microbiol.* 177 57–62. 10.1016/j.ijfoodmicro.2014.02.015 24607860

[B64] Wells-Bennik M. H. J. Eijlander R. T. den Besten H. M. W. Berendsen E. M. Warda A. K. Krawczyk A. O. (2016). Bacterial spores in food: survival, emergence, and outgrowth. *Annu. Rev. Food Sci. Technol.* 7 457–482. 10.1146/annurev-food-041715-033144 26934174

[B65] Wuytack E. Y. Boven S. Michiels C. W. (1998). Comparative study of pressure-induced germination of *Bacillus subtilis* spores at low and high pressures. *Appl. Environ. Microbiol.* 64 3220–3224. 9726863 10.1128/aem.64.9.3220-3224.1998 PMC106713

[B66] Zernike F. (1955). How I discovered phase contrast. *Science* 121 345–349. 10.1126/science.121.3141.345 13237991

[B67] Zhang P. Kong L. Wang G. Scotland M. Ghosh S. Setlow B. (2012). Analysis of the slow germination of multiple individual superdormant *Bacillus subtilis* spores using multifocus Raman microspectroscopy and differential interference contrast microscopy. *J. Appl. Microbiol.* 112 526–536. 10.1111/j.1365-2672.2011.05230.x 22212253

[B68] Zhang Y. F. Mathys A. (2019). Superdormant spores as a hurdle for gentle germination-inactivation based spore control strategies. *Front. Microbiol.* 9:e03163. 10.3389/fmicb.2018.03163 30662433 PMC6328458

[B69] Zhang Y. F. Moeller R. Tran S. Dubovcova B. Akepsimaidis G. Meneses N. (2018). *Geobacillus* and *Bacillus* spore inactivation by low energy electron beam technology: resistance and influencing factors. *Front. Microbiol.* 9:2720. 10.3389/fmicb.2018.02720 30532740 PMC6265500

