# Supplementary material for: Flow Cytometry Combined With Single Cell Sorting to Study Heterogeneous Germination of Bacillus Spores Under High Pressure
Source: Front Microbiol. 2020 Jan 21;10:3118. doi: 10.3389/fmicb.2019.03118 (PMC6985370; doi:10.3389/fmicb.2019.03118)
Supplement: Supplementary file 1 [file Data_Sheet_1.PDF]

Supplementary material

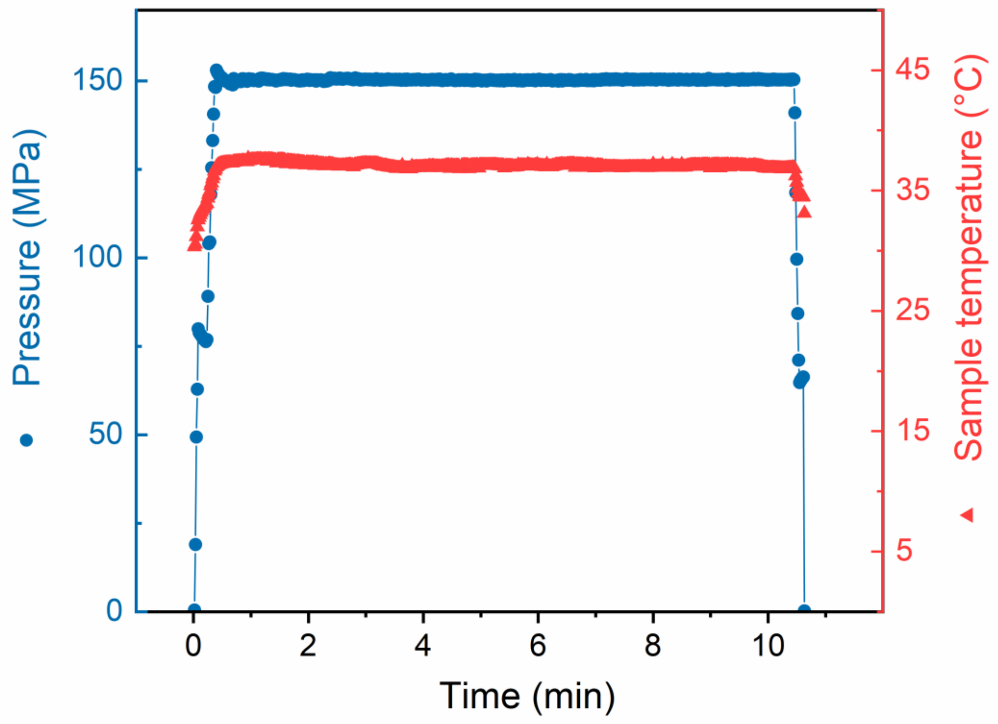

Supplementary Figure 1: Representative pressure/temperature profile for 10 min high pressure treated *Bacillus subtilis* spores at 150 MPa and 37°C. Pressure build-up and decompression rates were approx. 7 MPa/s and 12 MPa/s, respectively.
